# The impact of physical exercise with additional visual tasks on self-esteem in children: the mediating role of visual acuity

**DOI:** 10.3389/fpsyg.2025.1539278

**Published:** 2025-04-30

**Authors:** Guiming Zhu, Miyu Wang, Yuting Li, Pengfei Li, Haijie Shi, Limei Jiang, Sheng Zhou, Rongbin Yin

**Affiliations:** ^1^School of Physical Education, Soochow University, Suzhou, China; ^2^Department of Basic Course, Suzhou City University, Suzhou, China

**Keywords:** physical exercise, self-esteem, visual tasks, visual acuity, children aged 11–12

## Abstract

**Purpose:**

This study aimed to investigate the effects of physical exercise incorporating additional visual tasks on self-esteem and visual acuity in children aged 11–12. Specifically, it explored the relationship between self-esteem and visual acuity and examines whether visual acuity mediates the impact of such exercise on self-esteem.

**Methods:**

The study randomly selected four sixth-grade classes from a primary school in Suzhou as participants. The children were divided into two groups: one group engaged in physical exercise with additional visual tasks (*n* = 84), while the other group followed a regular physical exercise regimen (*n* = 83). The experiment lasted 16 weeks, and self-esteem levels, uncorrected distance visual acuity (UDVA), and kinetic visual acuity (KVA) were measured before and after the experiment.

**Results:**

Significant improvements in self-esteem were observed in both the experimental and control groups (*p* < 0.01). In the experimental group, notable enhancements were recorded in both UDVA and KVA for both eyes (*p* < 0.001). In contrast, the control group showed no significant change in left eye UDVA (*p* > 0.05), while right eye UDVA and KVA declined. A low positive correlation was identified between self-esteem and UDVA in both eyes within the experimental group, although no correlation was found between self-esteem and KVA. Additionally, left eye UDVA was moderately positively correlated with right eye UDVA. KVA was positively correlated with UDVA in both the left and right eyes. Physical exercise incorporating visual tasks was a significant positive predictor of self-esteem in 11-12-year-olds (*β* = 0.759, *p* < 0.01). UDVA in both eyes partially mediated the relationship between exercise and self-esteem (left eye 95% CI: [0.079, 0.400]; right eye 95% CI: [0.216, 0.666]).

**Conclusion:**

Physical exercise incorporating additional visual tasks can enhance self-esteem and improve both UDVA and KVA in children aged 11–12. Furthermore, the level of self-esteem in children was related to the level of UDVA in the right and left eyes. The UDVA of both eyes partially mediated the impact of physical exercise with additional visual tasks on self-esteem.

## 1 Introduction

During primary education, students’ self-awareness develops rapidly. At this stage, students became increasingly capable of recognizing their distinct characteristics compared to others, which can help them understand themselves better, gain self-experiences, and build self-esteem. Self-esteem is considered a personality trait that can predict an individual’s emotional state and life changes. It is not only a key element of psychological well-being but also a fundamental component of an individual’s self-system. The development of students’ self-esteem is closely linked to their mental health, significantly influencing their personal growth and social skills development (Rosenberg, 2001). Furthermore, self-esteem represents both an evaluative and emotional appraisal of oneself (Tian and Li, 2005), making it a valuable indicator in the academic and social development of children. Primary education serves as the foundation for students’ overall development, and children in this stage are at a pivotal point in self-esteem formation, characterized by high malleability (Coopersmith, 1967). Their mental health greatly affects their future academic achievements and personality development, which in turn impacts the overall development level of the nation and society. Children aged 11–12, who are typically in the upper grades of primary school, experience heightened self-awareness and an increased need for self-esteem. As such, fostering the development of self-esteem in this age group is of paramount importance and warrants significant attention.

The eyes are crucial organs for interacting with the external world. Currently, myopia among primary school students has become increasingly severe, garnering widespread concern across society. Studies showed that China has the highest rate of myopia in primary school-aged children globally, with an alarming trend of earlier onset. Therefore, it is urgent to prevent and control myopia in children and adolescents to protect their visual health. The visual development of children and adolescents has a sensitive period, during which their vision undergoes significant changes and is highly malleable. Thus, capitalizing on this sensitive period by incorporating school physical exercise along with myopia prevention mechanisms can help improve poor vision among primary school students (Li et al., 2023). Children and adolescents represent the future of national development, and society as a whole should support and promote their physical and mental health.

Physical exercise, as an active measure to prevent myopia, plays a vital role in promoting the healthy visual development of children and adolescents (Ong et al., 2018). Regular participation in physical activities can help protect children’s and adolescents’ vision by reducing eye fatigue associated with prolonged near work, enhancing ocular blood circulation, and improving overall visual health. Additionally, physical exercise contributes to general physical fitness by strengthening the body. During physical activities, the dynamic movement of the eyes and the constant changes in visual focus aid in relaxing the ciliary muscles, ocular muscles, and ligaments, thereby alleviating eye strain caused by close-up tasks (Abuleil et al., 2022).

Moreover, physical exercise is a key factor in maintaining and promoting mental health. It plays a positive role in improving students’ self-esteem, as increased physical activity can boost students’ self-esteem and, consequently, their psychological well-being (Ekeland et al., 2005). During physical exercise, students’ stress and anxiety are alleviated or eliminated, their sense of competition, team spirit, self-esteem, self-confidence, and perseverance are enhanced, and their ability to adapt to the social environment is improved (Liu et al., 2015). Through physical exercise, students can also experience the joy of success, which fosters a relaxed and happy state of mind.

At the same time, research had demonstrated a correlation between visual acuity and self-esteem, with visual acuity serving as a positive predictor of self-esteem (Maaswinkel et al., 2020). Specifically, individuals with better visual acuity tend to exhibit higher levels of self-esteem. The World Health Organization (WHO) of the United Nations underscored that health is not merely the absence of disease but also encompasses a state of complete physical, mental, and social well-being. Given that children and adolescents are the foundation of a nation’s future development, it is crucial to promote their overall health. Therefore, this study focused on the impact of physical activity on self-esteem and visual acuity in 11–12 year old children. Furthermore, it aimed to model the mediating role of visual acuity in the relationship between physical exercise and self-esteem.

## 2 Experimental subjects and methods

### 2.1 Experimental subjects

This study involved 167 children aged 11–12, randomly selected from four sixth-grade classes at a primary school in Suzhou. The participants were randomly assigned to either the experimental group (*n* = 84; 41 boys, 43 girls) or the control group (*n* = 83; 42 boys, 41 girls), with two classes in each group. The experimental group engaged in physical exercise incorporating additional visual tasks, while the control group participated in regular physical exercise. The intervention took place during the Physical Education and Health curriculum. Pre-experiment assessments revealed no significant differences between the two groups in terms of self-esteem, kinetic visual acuity, or uncorrected distance visual acuity, confirming their suitability for the study. The details can be found in Table 1.

**Table 1:** Descriptive statistical analysis of the experimental subjects (*N* = 167, M ± SD).

Indicator	Experimental group	Control group	*p*-value
Number of participants	84	83	
Self-esteem	31.536 ± 4.143	31.880 ± 5.235	0.639
UDVA (left eye)	4.756 ± 0.368	4.704 ± 0.363	0.356
UDVA (right eye)	4.666 ± 0.371	4.689 ± 0.346	0.670
KVA	0.389 ± 0.230	0.391 ± 0.234	0.963

The selection criteria were as follows: (1) Sixth-grade students enrolled in the selected primary school; (2) Students capable of participating in physical activities without major health conditions; (3) Students able to complete the experimental tasks without cognitive impairments; (4) Students who agreed to participate, with parental consent obtained prior to the experiment. The study protocol was in accordance with the Helsinki Declaration and was approved by the ethics committee of the first author’s university (No.SU-DA20201010H01). In this study, the informed consent form was distributed to the parents before the commencement of the experiment and the experimental procedure was agreed upon by the guardians of the subjects.

### 2.2 Experimental plan

Children aged 11–12 are in a critical period for both visual development and physical and mental growth. The experimental content was designed to align with the developmental characteristics of this age group, in accordance with the “Physical Education and Health Curriculum Standards for Compulsory Education (2022 Edition)” and the teaching objectives of the selected school. Basketball and soccer were chosen as the primary activities for the 16-week intervention, with 8 weeks dedicated to basketball training followed by 8 weeks of soccer training. Basketball training included ball-handling, dribbling, passing (both stationary and in motion), shooting, and tactical cooperation exercises. Similarly, soccer training comprised ball control, dribbling, passing (both stationary and in motion), shooting, and tactical cooperation exercises.

To meet the needs of the experiment, the learning of basketball and soccer skills was combined with ciliary muscle training principles. Specifically, static and dynamic visual targets, as well as far-to-near visual adjustment exercises (30 repetitions) (Yin et al., 2022) with a presentation time of 3 s (Zhou et al., 2023), were integrated into the basketball and soccer training plans. The curriculum was developed in line with the “health first” educational philosophy, ensuring that ciliary muscle training was seamlessly incorporated into the skill development activities. The design followed a student-centered approach, aiming to enhance core competencies in physical education and health while supporting both the physical and mental well-being of the students. The details can be found in Table 2.

**Table 2:** Design of the intervention program content.

Project	Exercise content	Exercise frequency	Exercise duration	Ciliary muscle adjustment training design
Basketball	Ball-handling exercises, stationary dribbling, dribbling on the move, stationary passing, passing on the move, shooting, tactical cooperation	3 sessions per week, 40 min per session	16 weeks in total	Based on the principles of ciliary muscle adjustment training, dynamic visual tasks that alternate between near and far vision were incorporated into the physical exercise. The control group had no ciliary muscle intervention and participated in regular physical education classes. In the experimental group, ciliary muscle adjustment training was integrated into each session, lasting 3 s per exercise, with a frequency of 30 to 60 repetitions.
Soccer	Ball-handling exercises, stationary dribbling, dribbling on the move, stationary passing, passing on the move, shooting, tactical cooperation			

Apart from the additional visual adjustment tasks, all groups were consistent in terms of exercise venues, duration, and activities to ensure the rigor of the experiment. The intervention spanned 16 weeks, with three 40-min sessions per week. Throughout the experiment, to minimize the influence of extraneous variables and ensure orderly progression, all physical education sessions were conducted by the same instructor, strictly adhering to the curriculum standards and the teaching plan. Uniformity was also upheld in terms of exercise content, intensity, density, and equipment, ensuring that external factors had minimal influence on the experimental outcomes.

### 2.3 Testing methods

Data on uncorrected distance visual acuity (UDVA), kinetic visual acuity (KVA), and self-esteem levels were collected both before the experiment (Week 1) and after the experiment (Week 17). The tests were conducted in a quiet, well-lit classroom within the experimental school, ensuring that the environment met the necessary testing standards. To minimize the influence of extraneous factors and enhance the accuracy and reliability of the results, several control measures were implemented. Prior to the testing, all personnel involved underwent professional training to familiarize themselves with the testing procedures and protocols. Before each test session, equipment was thoroughly inspected, and the surrounding environment and lighting were checked to ensure compliance with the required testing conditions.

#### 2.3.1 Self-esteem test

Self-esteem was measured using the Chinese version of the Rosenberg Self-Esteem Scale (SES), originally developed by Rosenberg and later translated and revised by Chinese scholars Ji Fuyi and Yu Xin. Considering cultural differences between Eastern and Western contexts, this study adopted the scoring adjustment proposed by Wang Ping, Gao Hua, et al., where the eighth item was positively scored. Although the SES was initially designed for adolescents, it has been extensively used across various age groups in China and is recognized as a valid and reliable instrument for self-esteem research. The scale used a Likert-type four-point scoring system, where 1 = “strongly disagree,” 2 = “disagree,” 3 = “agree,” and 4 = “strongly agree.” Six items (1, 2, 4, 6, 7, 8) are positively scored, while four items (3, 5, 9, 10) are reverse scored, resulting in a total score range of 10 to 40 points; higher scores indicate higher levels of self-esteem.

The self-esteem scale was tested for reliability and validity before the experimental intervention. The Cronbach’s coefficient of the self-esteem scale was 0.849 (>0.7), indicating good reliability of the scale, the KMO sampling aptitude measure was 0.840 (>0.7), and the Bartlett’s test of sphericity was significant at *p* < 0.001, demonstrating good validity.

#### 2.3.2 UDVA test

Uncorrected distance visual acuity (UDVA) was measured using a standard logarithmic visual acuity chart lightbox (GB11533—2011). The testing method and procedures strictly adhered to the standards set out in the “National Student Physical Fitness Health Survey Testing Protocol.” A marker was positioned 5 meters from the lightbox, and students were instructed to stand at this point while undergoing sequential testing for the left and right eyes. During the test, the non-tested eye was covered with an eye shield. Students who typically wore glasses were asked to remove them before testing. The lowest line correctly identified by each student was recorded as their UDVA.

#### 2.3.3 KVA test

Kinetic visual acuity (KVA) was assessed using the XP.14-TD-J905 kinetic visual acuity tester, produced by Shanghai Tuofeng Automation Technology Co., Ltd., in accordance with the national standard GB18463-2001 of the People’s Republic of China. Prior to testing, participants were given instructions on how to use the device. They were then tested sequentially based on their student numbers. Participants adjusted their seating height to align their eyes with the viewing aperture of the device. After ensuring that the participant was seated correctly and holding the joystick with their dominant hand, the test began with a card swipe. A “C”-shaped visual target appeared inside the device, simulating a dynamic approach from 50 meters at a speed of 30 km/h. The “C” gradually approached the participant’s eyes, with the gap in the “C” pointing in one of four directions: up, down, left, or right. Once the participant identified the direction of the gap, they quickly moved the joystick in the corresponding direction to complete the test. After each test, participants rested for 30 s. Each participant completed three trials, with the final KVA score being the average of the three. Scores ranged from 0.1 to 1.6, with higher scores indicating better kinetic visual acuity.

### 2.4 Statistical analysis

The test data were entered and organized using Excel 2019, and statistical analyses were conducted with SPSS 26.0. An independent samples t-test was used to assess the homogeneity of self-esteem and visual acuity scores between the experimental and control groups prior to the intervention. A repeated measures analysis of variance (ANOVA) was conducted to examine the main effects and interaction effects of group and time on self-esteem and visual acuity. If significant interaction effects were found, simple effects analysis was conducted. The chi-square test was used to compare the proportions of students with different levels of visual acuity before and after the experiment in both groups. An independent samples t-test was employed to compare the changes in self-esteem and visual acuity between the experimental and control groups before and after the intervention. Pearson correlation analysis was conducted to explore the relationships between the variables. Finally, the SPSS macro PROCESS, developed by Hayes, was used to test whether visual acuity mediated the effect of physical exercise with additional visual tasks on the self-esteem of sixth-grade students.

## 3 Results

### 3.1 Within-group and between-group comparisons of self-esteem and visual acuity in the experimental and control groups before and after the intervention

To investigate the impact of physical exercise with additional visual tasks on self-esteem and visual acuity in 11- to 12-year-old children, a 2 × 2 repeated measures ANOVA was conducted for uncorrected distance visual acuity (UDVA), kinetic visual acuity (KVA), and total self-esteem scores across both the experimental and control groups. The results can be presented in Tables 3, 4.

**Table 3:** Overall analysis of self-esteem and visual acuity in experimental and control groups before and after the intervention (M ± SD).

Indicator	Experimental group (*N* = 84)		Control group (*N* = 83)		Time effect	Between-group effect	Interaction effect
Time	Pre-test	Post-test	Pre-test	Post-test			
Self-Esteem	31.536 ± 4.143	34.202 ± 3.529	31.880 ± 5.235	32.615 ± 5.151	0.000**	0.363	0.000**
Left Eye UDVA	4.756 ± 0.363	4.854 ± 0.344	4.704 ± 0.363	4.730 ± 0.371	0.000**	0.112	0.001**
Right Eye UDVA	4.666 ± 0.371	4.791 ± 0.358	4.689 ± 0.346	4.674 ± 0.367	0.000**	0.397	0.000**
KVA	0.389 ± 0.230	0.447 ± 0.221	0.391 ± 0.234	0.369 ± 0.241	0.084	0.256	0.000**

** indicates *p* < 0.01, representing a highly significant difference.

**Table 4:** Simple effect analysis of self-esteem and visual acuity in experimental and control groups before and after the intervention.

Indicator	Source of variation	*F*-value	*p*-value
Self-Esteem	Experimental group pre- and post-test	107.266	0.000**
	Control group pre- and post-test	8.051	0.005**
	Post-test comparison between experimental and control groups	5.419	0.021*
Left Eye UDVA	Experimental group pre- and post-test	44.906	0.000**
	Control group pre- and post-test	3.271	0.072
	Post-test comparison between experimental and control groups	4.970	0.027*
Right Eye UDVA	Experimental group pre- and post-test	81.197	0.000**
	Control group pre- and post-test	1.260	0.263
	Post-test comparison between experimental and control groups	4.345	0.039*
KVA	Experimental group pre- and post-test	15.210	0.000**
	Control group pre- and post-test	2.023	0.157
	Post-test comparison between experimental and control groups	4.773	0.030*

* indicates *p* < 0.05, representing a significant difference; ** indicates *p* < 0.01, representing a highly significant difference.

A 2 (Time: Pre-test, Post-test) × 2 (Group: Experimental, Control) repeated measures ANOVA was applied to the total scores from the Rosenberg Self-Esteem Scale (SES) at pre-test and post-test. The results revealed a significant main effect of Time (*p* < 0.001), while the main effect of Group was not significant (*p* = 0.363). However, a significant Time×Group interaction effect was observed (*p* < 0.001). A simple effects analysis showed a significant Time effect on self-esteem within the experimental group (*F* (1, 165) = 107.266, *p* < 0.001), indicating a significant difference in self-esteem before and after the intervention in the experimental group. In the control group, the simple effect of Time was also significant (*F* (1, 165) = 8.051, *p* = 0.005), indicating a significant difference in self-esteem before and after the intervention. Additionally, the simple effect of Group at the post-test was significant (*F* (1, 165) = 5.419, *p* = 0.021), indicating a significant difference in self-esteem between the experimental and control groups after the intervention.

A 2 (Time: Pre-test, Post-test) × 2 (Group: Experimental, Control) repeated measures ANOVA was conducted to analyze left-eye uncorrected distance visual acuity (UDVA). The results showed a significant main effect of Time (*p* < 0.001) for left-eye UDVA, but no significant main effect of Group (*p* = 0.112). A significant Time×Group interaction effect was observed (*p* = 0.001). Simple effects analysis revealed a significant simple effect of Time for left-eye UDVA in the experimental group (*F* (1, 165) = 44.906, *p* < 0.001), indicating a significant difference in left-eye UDVA before and after the intervention in the experimental group. However, no significant Time effect was found in the control group (*F* (1, 165) = 3.271, *p* = 0.072), indicating no notable change. However, a significant Group effect at post-test (*F* (1, 165) = 4.970, *p* = 0.027) demonstrated a significant difference in left-eye UDVA between the experimental and control groups.

For right-eye uncorrected distance visual acuity (UDVA), a 2 (Time: Pre-test, Post-test) × 2 (Group: Experimental, Control) repeated measures ANOVA revealed a significant main effect of Time (*p* < 0.001), but no significant main effect of Group (*p* = 0.397). The Time×Group interaction effect was significant (*p* < 0.001). Simple effects analysis showed a significant simple effect of Time for right-eye UDVA in the experimental group (*F* (1, 165) = 81.197, *p* < 0.001), indicating a significant difference in right-eye UDVA before and after the intervention in the experimental group. However, no significant Time effect was observed in the control group (*F* (1, 165) = 1.260, *p* = 0.263). A significant Group effect at the post-test (*F* (1, 165) = 4.345, *p* = 0.039) indicated a notable difference in right-eye UDVA between the experimental and control groups following the intervention.

For kinetic visual acuity (KVA), a 2 × 2 repeated measures ANOVA found no significant main effect of Time (*p* = 0.084) or Group (*p* = 0.256). However, the Time×Group interaction effect was significant (*p* < 0.001). Simple effects analysis indicated a significant simple effect of Time for KVA in the experimental group (*F* (1, 165) = 15.210, *p* < 0.001), indicating a significant difference in KVA before and after the intervention in the experimental group. No significant Time effect was found in the control group (*F* (1, 165) = 2.023, *p* = 0.157). Additionally, the Group effect at the post-test was significant (*F* (1, 165) = 4.773, *p* = 0.030), highlighting a significant difference in KVA between the experimental and control groups after the intervention.

### 3.2 Correlation between self-esteem, uncorrected distance visual acuity, and kinetic visual acuity in the experimental group

Pearson correlation analysis was performed to examine the relationships between self-esteem, uncorrected distance visual acuity (UDVA) in both eyes, and kinetic visual acuity (KVA) in the experimental group. As shown in Table 5, a significant low positive correlation was found between self-esteem and UDVA in the left eye (*p* < 0.01, *r* = 0.300) and in the right eye (*p* < 0.01, *r* = 0.329). However, no significant correlation was observed between KVA and self-esteem (*p* > 0.05). A significant moderate positive correlation was found between UDVA in the left and right eyes (*p* < 0.01, *r* = 0.656). Additionally, a significant low positive correlation was observed between KVA and UDVA in the left eye (*p* < 0.01, *r* = 0.324), and a significant moderate positive correlation between KVA and UDVA in the right eye (*p* < 0.01, *r* = 0.476).

**Table 5:** Correlation coefficients between self-esteem and visual acuity in the experimental group after the intervention (*N* = 84).

Indicator	Self-esteem	Left eye UDVA	Right eye UDVA	KVA
Self-esteem	1			
Left eye UDVA	0.300^**^	1		
Right eye UDVA	0.329^**^	0.656^**^	1	
KVA	0.934	0.324^**^	0.476^**^	1

* indicates *p* < 0.05, representing a significant difference; ** indicates *p* < 0.01, representing a highly significant difference.

### 3.3 Mediation effect of uncorrected distance visual acuity on the relationship between physical exercise with additional visual tasks and self-esteem

#### 3.3.1 Regression analysis of physical exercise with additional visual tasks, uncorrected distance visual acuity, and self-esteem

To evaluate the hypothesized mediation model, the data were analyzed accordingly. Based on the correlation analysis, physical exercise with additional visual tasks was used as the independent variable, the difference in uncorrected distance visual acuity (UDVA) between pre- and post-intervention for both eyes served as the mediating variable, and the difference in self-esteem score was used as the dependent variable. Mediation analysis was conducted using the Process macro for SPSS, Model 4. The results can be presented in Table 6.

**Table 6:** Regression analysis of physical exercise with additional visual tasks, uncorrected distance visual acuity, and self-esteem.

Regression equation		Overall fit index				
Predictor variables	Result variables	*R*	*R* ^2^	SE	*t*	*β*
Physical exercise with additional visual tasks	Self-Esteem	0.381	0.145	0.144	5.290^**^	0.759
Physical exercise with additional visual tasks	Left Eye UDVA	0.259	0.021	0.207	3.442^**^	0.516
	Right Eye UDVA	0.486	0.237	0.136	7.149^**^	0.970
Physical exercise with additional visual tasks	Self-Esteem	0.575	0.331	0.132	4.012^**^	0.529
Left eye UDVA				0.066	6.742^**^	0.446
Physical exercise with additional visual tasks	Self-Esteem	0.545	0.297	0.149	2.188^*^	0.327
Right eye UDVA				0.075	5.949^**^	0.446

* indicates *p* < 0.05, representing a significant difference; ** indicates *p* < 0.01, representing a highly significant difference.

A linear regression analysis was conducted with physical exercise with additional visual tasks as the independent variable and self-esteem score (difference) as the dependent variable. The results of the regression equation showed a significant difference in the regression equation with *β* = 0.759, *t* = 5.290, *p* < 0.01, indicating that physical exercise with additional visual tasks significantly and positively predicted self-esteem.

A linear regression analysis was conducted with physical exercise with additional visual tasks as the independent variable and UDVA in the left eye as the dependent variable. The results of the regression equation showed a significant difference in the regression equation with *β* = 0.516, *t* = 3.442, *p* < 0.01, indicating that physical exercise with additional visual tasks significantly and positively predicted UDVA in the left eye. A linear regression analysis was conducted with physical exercise with additional visual tasks and UDVA in the left eye as independent variables and self-esteem score (difference) as the dependent variable. The results of the regression analysis of physical exercise with additional visual tasks and self-esteem score (difference) showed a significant difference in the regression equation with *β* = 0.529, *t* = 4.012, *p* < 0.01, indicating that physical exercise with additional visual tasks still positively predicted self-esteem. The results of the regression analysis of UDVA in the left eye and self-esteem scores (difference) showed a significant difference in the regression equation with *β* = 0.446, *t* = 6.742, *p* < 0.01, indicating that UDVA in the left eye positively predicted self-esteem.

A linear regression analysis was conducted with physical exercise with additional visual tasks as the independent variable and UDVA in the right eye as the dependent variable. The results of the regression equation showed a significant difference in the regression equation with *β* = 0.970, *t* = 7.149, *p* < 0.01, indicating that physical exercise with additional visual tasks significantly and positively predicted UDVA in the right eye. A linear regression analysis was conducted with physical exercise with additional visual tasks and UDVA in the right eye as independent variables and self-esteem score (difference) as dependent variable. The results of the regression analysis of physical exercise with additional visual tasks and self-esteem score (difference) showed a significant difference in the regression equation with *β* = 0.327, *t* = 2.188, *p* < 0.05, indicating that physical exercise with additional visual tasks still positively predicted self-esteem. The results of the regression analysis of UDVA in the right eye and self-esteem score (difference) showed a significant difference in the regression equation with *β* = 0.446, *t* = 5.949, *p* < 0.01, indicating that UDVA in the right eye positively predicted self-esteem.

#### 3.3.2 Mediation effect of uncorrected distance visual acuity in the relationship between physical exercise with additional visual tasks and self-esteem

**Figure 1:**
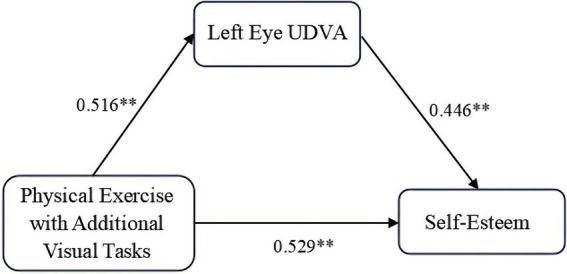
Path diagram of regression coefficients for mediation effect of left eye uncorrected distance visual acuity. ** indicates *p* < 0.01, representing a highly significant difference.

The Bootstrap method was employed to assess the mediation effect of uncorrected distance visual acuity (UDVA) in the left eye on the relationship between physical exercise with additional visual tasks and self-esteem. As presented in Table 7, the direct effect of physical exercise with additional visual tasks on self-esteem was significant (*β* = 0.529), accounting for 69.70% of the total effect. Additionally, the mediation effect of left-eye UDVA was significant (*β* = 0.230), accounting for 30.30% of the total effect, indicating that UDVA in the left eye partially mediated the relationship between physical exercise with additional visual tasks and self-esteem. The effect size’s 95% confidence interval was estimated by resampling the data 5,000 times. The results confirmed that the direct effect of physical exercise with additional visual tasks on self-esteem, the indirect effect via left-eye UDVA, and the total effect were all significant, with the 95% confidence interval excluding zero. This confirmed that the mediation model constructed in this study is valid, as illustrated in Figure 1.

**Table 7:** Mediation effect of left eye uncorrected distance visual acuity tested by bootstrap method.

Type of effect	Effect value	95% Confidence interval		Effect value proportion
		LLCI	ULCL	
Total effect	0.759	1.211	2.653	100%
Direct effect	0.529	0.684	2.009	69.70%
Indirect effect	0.230	0.079	0.400	30.30%

**Figure 2:**
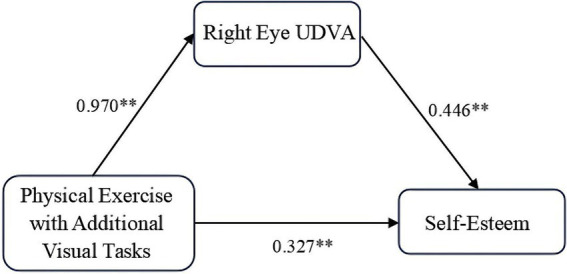
Path diagram of regression coefficients for mediation effect of right eye uncorrected distance visual acuity. ** indicates *p* < 0.01, representing a highly significant difference.

Similarly, the Bootstrap method was applied to assess the mediation effect of UDVA in the right eye on the relationship between physical exercise with additional visual tasks and self-esteem. As shown in Table 8, the direct effect of physical exercise with additional visual tasks on self-esteem was significant (*β* = 0.327), accounting for 43.08% of the total effect. Moreover, the mediation effect of right-eye UDVA was also significant (*β* = 0.432), accounting for 56.92% of the total effect, indicating that UDVA in the right eye partially mediated the relationship between physical exercise with additional visual tasks and self-esteem. The 95% confidence interval for the effect size was calculated using 5,000 bootstrap resamples. The results confirmed that the direct effect of physical exercise with additional visual tasks on self-esteem, the indirect effect through the right eye’s UDVA, and the total effect were all significant, with the 95% confidence interval excluding zero. This confirmed the validity of the mediation model constructed in this study, as depicted in Figure 2.

**Table 8:** Mediation effect of right eye uncorrected distance visual acuity tested by bootstrap method.

Type of effect	Effect value	95% Confidence interval		Effect value proportion
		LLCI	ULCI	
Total effect	0.759	0.476	1.043	100%
Direct effect	0.327	0.081	1.583	43.08%
Indirect effect	0.432	0.216	0.666	56.92%

## 4 Discussion

### 4.1 Developmental status of self-esteem and visual acuity in 11–12 year old children

#### 4.1.1 Development of self-esteem in 11–12 year old children

Self-esteem levels change with age, with self-esteem showing higher levels in childhood and a downward trend beginning in late childhood and continuing throughout adolescence (Robins et al., 2002). The ages of 11–12 are in the transition between childhood and adolescence and are also a critical time for the development of self-esteem (Zhang et al., 2010). At this stage, children are very sensitive to the judgments of others and are prone to paranoia and vacillations, while physiological changes and social and academic pressures can make their self-esteem levels extremely unstable. Therefore, it is particularly important to help children develop good levels of self-esteem during the 11–12 year old period. The study revealed that the average self-esteem score of children in the experimental group was 31.536, while the control group scored 31.880, indicating that the self-esteem levels of 11- to 12-year-old children in this study were within the normal range. This result aligns with Sun’s (2022) research, which found an average score of 31.18 for 453 upper elementary school students. As children advance through elementary school, their self-awareness and ability to self-evaluate improve, leading to a heightened need for self-esteem and confidence. The desire for positive feedback and praise from others also intensifies (Tuttle, 1987), contributing to a rise in self-esteem levels. Studies have shown that self-esteem can significantly influence academic performance and self-efficacy (Mihaela, 2015). Children with positive self-esteem tend to exhibit more stable emotions and greater confidence in completing academic tasks, thus enhancing their academic self-efficacy. Therefore, fostering self-esteem in children is essential for promoting academic achievement and supporting their future development.

#### 4.1.2 Development of visual acuity in 11–12 year old children

The results of this study revealed that the average uncorrected distance visual acuity of the left and right eyes in the experimental group was 4.756 and 4.666, respectively, while in the control group, the averages were 4.704 and 4.689, respectively. The average kinetic visual acuity was 0.389 in the experimental group and 0.391 in the control group. These findings suggested that the visual health of 11- to 12-year-old children is concerning and requires urgent attention and preventive measures. Myopia has emerged as a major health issue affecting the development of children and adolescents in China, with consistently high prevalence rates. According to the Eighth National Student Physical Fitness Survey, the incidence of poor vision and myopia among school-aged children remains alarmingly high. The findings of this study underscore the urgent need for targeted vision prevention and intervention measures, as both uncorrected distance visual acuity and kinetic visual acuity among children in this age group are below optimal levels.

### 4.2 The effect of physical activity with additional visual tasks on self-esteem and visual acuity in 11–12 year old children

#### 4.2.1 The effect of physical activity with additional visual tasks on self-esteem in 11–12 year old children

There was a significant positive correlation between physical activity and self-esteem (Zheng et al., 2022), and physical activity had a direct positive effect on individual self-esteem levels (Guo et al., 2017). Engagement in appropriate physical activities can provide students with positive physical and psychological experiences (Dong and Yu, 2024), playing a crucial role in fostering self-esteem and confidence among adolescents. The intervention in this study led to improvements in self-esteem scores for both the experimental and control groups, suggesting that physical exercise positively influences children’s self-esteem. This aligns with findings from Knox and Muros (2017), who reported that individuals regularly engaged in physical activity tend to maintain higher self-esteem and a more positive psychological state.

Both the mastery hypothesis and the social reinforcement theory suggest that physical activity can have a positive impact on self-esteem. The mastery hypothesis is part of a social cognitive theory that suggests that exercise improves physical functioning and skills, and encourages individuals to increase their self-efficacy. Social reinforcement theory suggests that family and friends improve exercisers’ self-perceptions by praising and encouraging them, demonstrating that interacting with others during physical activity can play a role in their self-esteem. In sports, elementary school students are required to collaborate and interact with their peers and groups, thereby increasing their individual teamwork and collective respect during sports participation (Bu et al., 2022), which in turn increases their level of self-esteem. The research showed that sports such as badminton and basketball had a significant effect on the level of self-esteem of the students (Zhu and Yan, 2006), and that moderate-intensity exercise significantly increased the level of physical self-esteem (Wang, 2016). The intervention in this study included basketball and soccer, both of which emphasize cooperation, likely promoted teamwork and increased students’ interest in sports, leading to improvements in psychological well-being and self-esteem.

The results of this study indicated that the experimental group experienced significantly greater improvements in self-esteem compared to the control group, demonstrating the effectiveness of the intervention in enhancing self-esteem. There was a correlation between the level of self-esteem and the level of vision, with better vision leading to higher levels of self-esteem. Visual acuity also plays a critical role in balance, spatial judgment, reaction time, coordination, concentration, and confidence (Sun, 2003). Physical exercise with additional visual tasks enables students to strengthen the ciliary muscles of distance-vision and near-vision, exercise eye muscles and regulation, and improve vision levels, which in turn has a positive effect on self-esteem and promotes healthy psychological development in children.

#### 4.2.2 The effect of physical exercise with additional visual tasks on UDVA in 11–12 year old children

Children’s visual function and eye health are closely linked to physical activity. The results of this study indicated that UDVA in both eyes improved in both the control and experimental groups after the intervention, with a significantly greater improvement observed in the left eye of the experimental group. While the experimental group also showed improvement in right-eye UDVA, the control group experienced a slight decline. Research suggested that physical exercise causes continuous contraction of the eye muscles, allowing the ciliary muscles to relax under prolonged fatigue and pressure, thereby improving UDVA (Hu et al., 2009). Additionally, outdoor light exposure increases dopamine release, which inhibits axial elongation of the eye (Norton and Siegwart, 2013). During physical exercise, reduced sedentary behavior and varying distances in outdoor activities minimize the risk of visual strain (Yurova et al., 2017), while reducing harmful eye habits and improving visual function.

The addition of visual tasks to physical exercise, involving near-to-far ciliary muscle training, allows for relaxation of both the ciliary and periocular muscles during practice, thereby enhancing the effectiveness (Jonas et al., 2016). However, differences in sleep duration and visual habits among students may account for the slight decline in UDVA for the right eye in the control group. Studies have shown that only 12.50% of 11–12 year old children achieve the recommended 10 h of daily sleep (Zhou et al., 2015), as stipulated by the Ministry of Education, and these differences can influence the effectiveness of physical exercise in preventing myopia (Li et al., 2023).

#### 4.2.3 The effect of physical exercise with additional visual tasks on KVA in 11–12 year old children

The results of this study indicated an improvement in KVA in the experimental group, while a slight decline was observed in the control group. Research indicated that the developmental window for KVA is between the ages of 8 and 14, and the ciliary muscle training intervention in this study led to improvements in the KVA of elementary school students (Zhou et al., 2020). The study confirmed that physical exercise with additional visual tasks enhances KVA by improving students’ ability to adjust their vision, allowing for better discrimination of moving objects and the accumulation of dynamic visual experiences. Given the increasing academic pressures faced by 11–12 year old children, and the heightened risk of myopia during this critical developmental stage, implementing early vision prevention and control measures in elementary schools is essential for optimal visual outcomes.

### 4.3 Relationship between self-esteem, UDVA, and KVA in 11–12 year old children

The results of this study indicated a significant correlation between self-esteem, UDVA in both eyes, and KVA in the experimental group. Notably, a positive relationship exists between UDVA and self-esteem. According to Shavelson’s hierarchical model of self-concept, participation in physical activities enhances the physical self-concept, which in turn improves overall self-concept and self-esteem. Previous research has shown that wearing glasses due to myopia can negatively affect self-esteem, particularly when glasses are first introduced during childhood or adulthood, more so than during adolescence (Terry et al., 1983). The use of corrective lenses often leads to a negative body image, reducing physical self-esteem and, subsequently, overall self-esteem. Myopia can also foster feelings of loneliness, further lowering self-esteem and leading to social challenges (You et al., 2022). Since self-worth during childhood and adolescence is strongly influenced by peer evaluations, myopic children may perceive themselves more negatively. Moreover, physical exercise has been found to improve body image and perceived physical competence, which indirectly enhances overall self-esteem (He and Zhang, 2002). Visual acuity can affect sports performance (Zurita-Ortega et al., 2017), which in turn influences physical self-esteem and overall self-esteem.

The results of this study showed that the left eye UDVA was significantly and positively correlated with the right eye UDVA in the experimental group. In the experimental group, KVA was significantly positively correlated with UDVA in both the left and right eyes. Research indicated that better KVA is associated with improvements in UDVA, as KVA reflects the accommodative function of the ciliary muscle, which can predict the level of UDVA to some extent. The positive relationship between UDVA and KVA suggests a consistent role of ciliary muscle function in both (Cao et al., 2019). Consequently, interventions that improve KVA through physical activity may also contribute to enhancements in UDVA.

### 4.4 The mediating role of uncorrected distance visual acuity in the impact of physical exercise with additional visual tasks on self-esteem

The results of this study indicated that UDVA in both eyes partially mediates the effect of physical exercise with additional visual tasks on self-esteem in 11–12 year old children. In other words, the influence of physical exercise with additional visual tasks on self-esteem is partially transmitted through its impact on uncorrected distance visual acuity.

Physical exercise is inherently a social process that involves teamwork, which enhances an individual’s sense of team spirit and collective honor, thereby boosting self-esteem. The onset of myopia may affect learning and daily life, leading to changes in both physical and mental well-being (Yang and Zhang, 2005). Furthermore, the need for participation in physical exercise not only improves physical self-concept but also increases social interaction, thereby enhancing both overall self-concept and self-esteem (Rosenberg, 2001).

Poor visual acuity can negatively affect academic performance and, if left uncorrected, can impair psychological well-being and long-term development. Research shows that children experiencing significant visual symptoms, such as eye strain, tend to have lower self-assessments of their physical appearance, academic abilities, social interactions, behavior, and overall self-worth. If these visual symptoms impact self-perception, alleviating them could lead to a more positive self-assessment (Liu et al., 2015). Additionally, there is a significant positive correlation between physical activity, physical self-esteem, and overall self-esteem (Dias et al., 2002). Physical activity improves physical self-esteem, which subsequently enhances overall self-esteem (Sun and Zhang, 2020).

Myopia is a societal concern that is closely linked to psychological health. Addressing this issue requires coordinated efforts from students, parents, schools, and the broader community to implement effective prevention and control measures. Incorporating ciliary muscle training and visual tasks into physical education and health programs can help control and improve visual acuity, thereby enhancing self-esteem and promoting overall health and well-being.

## 5 Conclusion

Physical exercise incorporating additional visual tasks can enhance self-esteem and improve both UDVA and KVA in children aged 11–12. Furthermore, the level of self-esteem in children was related to the level of UDVA in the right and left eyes. The UDVA of both eyes partially mediated the impact of physical exercise with additional visual tasks on self-esteem.

## 6 Limitations and future directions

The sample size in this study was limited. Due to the study’s long-term and complex nature, only a subset of sixth-grade students was included. Future studies should aim to include larger and more diverse samples to increase generalizability.

This study focused exclusively on uncorrected distance visual acuity and kinetic visual acuity as measures of visual function, neglecting other important ocular parameters. Future research should explore additional factors such as axial length, anterior chamber depth, corneal curvature, refractive error, and intraocular pressure to provide a more comprehensive assessment of visual health.

## Data Availability

The original contributions presented in the study are included in the article/supplementary material, further inquiries can be directed to the corresponding authors.

## References

[ref1] Abuleil D. Thompson B. Dalton K. (2022). Aerobic exercise and human visual cortex neuroplasticity: a narrative review. Neural Plast. 2022:6771999. doi: 10.1155/2022/6771999, PMID: 35915651 PMC9338869

[ref2] Bu T. Li J. M. Min S. C. (2022). Philosophical connotation and era value of the new concept of cultivating people by virtue in physical education in the new era. J. Beijing Sport Univ 45, 96–107. doi: 10.19582/j.cnki.11-3785/g8.2022.06.009

[ref3] Cao J. Y. Cai G. Wang G. X. Yin R. B. Sun L. (2019). Effects of physical activities with visual tasks on kinetic and static visual acuity in children. Chinese J. Rehab. Theory Prac. 25, 112–115. doi: 10.3969/j.issn.1006-9771.2019.01.016

[ref4] Coopersmith S. (1967). The antecedents of self-esteem. San Francisco: W.H.Freeman & Co Ltd.

[ref5] Dias L. Manny R. E. Hyman L. Fern K. Correction of Myopia Evaluation Trial Group (2002). The relationship between self-esteem of myopic children and ocular and demographic characteristics. Optom. Vision Sci. 79, 688–696. doi: 10.1097/00006324-200211000-00006, PMID: 12462537

[ref6] Dong P. Yu S. M. (2024). Interdisciplinary theme learning of physical education and health courses based on Core literacy: connotation determination, design process and promotion strategy. J. Tianjin Univ. Sport 39, 56–63. doi: 10.13297/j.cnki.issn1005-0000.2024.01.009

[ref7] Ekeland E. Heian F. Hagen K. B. Abbott J. Nordheim L. (2005). Exercise to improve self-esteem in children and young people. Campbell Syst. Rev. 1, 1–52. doi: 10.4073/csr.2005.4 PMC12935395 14974029

[ref8] Guo Q. G. Luo J. Sang M. L. Xie H. D. (2017). Influence of self-esteem and body image on sports participation in students. J. Xi'an Physical Educ. Univ. 34, 730–738. doi: 10.16063/j.cnki.issn1001-747x.2017.06.015

[ref9] He L. Zhang L. W. (2002). Relationship between evaluation modes of abstract body self-esteem, concrete body self-esteem and life satisfaction. J. Beijing Sport Univ. 2002:320-323+330. doi: 10.19582/j.cnki.11-3785/g8.2002.03.012

[ref10] Hu Y. N. Chu R. Y. Lv F. Qu J. (2009). Myopic eyes: People’s Medical Publishing House. 204.

[ref11] Jonas J. B. Xu L. Wei W. B. Wang Y. X. Jiang W. J. Bi H. S.. (2016). Myopia in China: a population-based cross-sectional, histological, and experimental study. Lancet 388:S20. doi: 10.1016/s0140-6736(16)31947-x

[ref12] Knox E. Muros J. J. (2017). Association of lifestyle behaviours with self-esteem through health-related quality of life in Spanish adolescents. Eur. J. Pediatr. 176, 621–628. doi: 10.1007/s00431-017-2886-z, PMID: 28265762 PMC5415583

[ref13] Li J. P. Chen C. H. Li S. J. (2023). Prevention and control strategies of school sports based on vision monitoring in primary school students. Zhejiang Sport Sci. 45, 93–98. doi: 10.3969/j.issn.1004-3624.2023.04.016

[ref14] Liu M. Wu L. Ming Q. (2015). How does physical activity intervention improve self-esteem and self-concept in children and adolescents? Evidence from a Meta-Analysis. PloS One 10:e0134804. doi: 10.1371/journal.pone.0134804, PMID: 26241879 PMC4524727

[ref15] Maaswinkel I. M. Van Der Aa H. P. A. van Rens G. H. Beekman A. T. Twisk J. W. van Nispen R. M. (2020). Mastery and self-esteem mediate the association between visual acuity and mental health: a population-based longitudinal cohort study. BMC Psychiatry 20:461. doi: 10.1186/s12888-020-02853-0, PMID: 32972387 PMC7513319

[ref16] Mihaela P. (2015). Psychological factors of academic success. Procedia Soc. Behav. Sci. 180, 1632–1637. doi: 10.1016/j.sbspro.2015.02.318

[ref17] Norton T. T. Siegwart J. T. (2013). Light levels, refractive development, and myopia-a speculative review. Exp. Eye Res. 114, 48–57. doi: 10.1016/j.exer.2013.05.004, PMID: 23680160 PMC3742693

[ref18] Ong S. R. Crowston J. G. Loprinzi P. D. Ramulu P. Y. (2018). Physical activity, visual impairment, and eye disease. Eye 32, 1296–1303. doi: 10.1038/s41433-018-0081-8, PMID: 29610523 PMC6085324

[ref19] Robins R. W. Trzesniewski K. H. Tracy J. L. Gosling S. D. Potter J. (2002). Global self-esteem across the life span. Psychol. Aging 17, 423–434. doi: 10.1037/0882-7974.17.3.423, PMID: 12243384

[ref20] Rosenberg S. A. (2001). Progress in human tumour immunology and immunotherapy. Nature 411, 380–384. doi: 10.1038/35077246, PMID: 11357146

[ref21] Sun Y. L. (2003). The discussion on basketball Player's visual ability and its training. J. Chengdu Sport Univ. 2003, 49–51. doi: 10.3969/j.issn.1001-9154.2003.02.013

[ref22] Sun Y. N. (2022). Influence of self-esteem on life satisfaction of middle and senior primary school students and its intervention. Central China Normal Univ. doi: 10.27159/d.cnki.ghzsu.2022.000324

[ref23] Sun C. Zhang G. L. (2020). The impact of physical activities on Adolescents' interpersonal competence: a chain mediating model. Chinese J. Sports Med. 39, 47–52. doi: 10.16038/j.1000-6710.2020.01.009

[ref24] Terry R. L. Berg A. J. Phillips P. E. (1983). The effect of eyeglasses on self-esteem. J. Am. Optom. Assoc. 54, 947–949. 6630845

[ref25] Tian L. M. Li S. (2005). Differentiating and analyzing the concept of self-esteem. Psychol. Explor. 2, 26–29. doi: 10.3969/j.issn.1003-5184.2005.02.006

[ref26] Tuttle D. W. (1987). The role of the special education teacher-counselor in meeting students’ self-esteem needs. J. Visual Impair. Blind. 81, 156–161. doi: 10.1177/0145482X8708100409

[ref27] Wang Y. J. (2016). Comparative analysis of moderate-intensity physical exercise on college students' physical self-esteem and psychological capital. Chin. J. School Health 37, 1661–1663. doi: 10.16835/j.cnki.1000-9817.2016.11.019

[ref28] Yang L. Z. Zhang L. H. (2005). A study on the structure of self-esteem in children aged from 3 to 9. J. Psychol. Sci 28, 23–27. doi: 10.16719/j.cnki.1671-6981.2005.01.006

[ref29] Yin R. Xu J. Wang H. Zhou S. Zhang M. Cai G. (2022). Effect of physical activity combined with extra ciliary-muscle training on visual acuity of children aged 10-11. Front. Public Health 10:949130. doi: 10.3389/fpubh.2022.949130, PMID: 36111187 PMC9468474

[ref30] You L. J. Hui Y. N. Zhang L. (2022). Research methods and advances in the impact of myopia on adolescent mental health. Intern. Eye Sci. 22, 1827–1831. doi: 10.3980/j.issn.1672-5123.2022.11.13

[ref31] Yurova O. V. Andjelova D. V. Chayka A. A. (2017). The influence of physical loads on the functional parameters of the eyes in the children and adolescents regularly engaged in sports activities. Vopr. Kurortol. Fizioter. Lech. Fiz. Kult. 94, 44–48. doi: 10.17116/kurort201794344-48, PMID: 28884738

[ref32] Zhang Y. X. Re N. Wei H. Zhang X. R. Chen J. Gu C. H. (2010). Self-esteem across the life span. Adv. Psychol. Sci. 18, 1128–1135.

[ref33] Zheng Y. Yan J. Zhu H. Zhu F. S. Cheng X. Y. (2022). Improving college students' interpersonal relationship through basketball: mediating effect of self-contro. Chin. J. Health Psychol. 30, 465–471. doi: 10.13342/j.cnki.cjhp.2022.03.029

[ref34] Zhou Z. Morgan I. G. Chen Q. Jin L. He M. Congdon N. (2015). Disordered sleep and myopia risk among Chinese children. PloS One 10:e0121796. doi: 10.1371/journal.pone.0121796, PMID: 25811755 PMC4374782

[ref35] Zhou S. Zhang M. Zheng W. Yin R. Chen G. (2023). Effects of physical activity combined with different visual target presentation durations of ciliary-muscle training on visual acuity in children. Front. Public Health 11:1191112. doi: 10.3389/fpubh.2023.1191112, PMID: 37538276 PMC10394291

[ref36] Zhou S. Zhou C. Tan Q. Qiu F. B. Cai G.. (2020). Effect of closed skills physical activity exercises with dynamic visual task on visual function for pupils with myopia at grade four at primary school. Chinese J. Rehab. Theory Prac. 26, 1383–1389. doi: 10.3969/j.issn.1006-9771.2020.12.003

[ref37] Zhu F. S. Yan J. (2006). Effects of basketball activity on self-esteem and mental health among male college students [J]. Chin. J. School Health 7, 581–582. doi: 10.3969/j.issn.1000-9817.2006.07.025

[ref38] Zurita-Ortega F. Castro-Sánchez M. Rodríguez-Fernández S. Cofré-Boladós C. Chacón-Cuberos R. Martínez-Martínez A.. (2017). Physical activity, obesity and self-esteem in chilean schoolchildren. Rev. Med. Chile 145, 299–308. doi: 10.4067/s0034-98872017000300006, PMID: 28548189

